# PROSPECT guideline for haemorrhoid surgery: A systematic review and procedure-specific postoperative pain management recommendations

**DOI:** 10.1097/EA9.0000000000000023

**Published:** 2023-05-26

**Authors:** Alexis Bikfalvi, Charlotte Faes, Stephan M. Freys, Girish P. Joshi, Marc Van de Velde, Eric Albrecht

**Affiliations:** From the Department of Anaesthesia, University Hospital of Lausanne and University of Lausanne, Lausanne, Switzerland (AB, EA), the Department of Cardiovascular Sciences and Department of Anaesthesia, University Hospitals of the KU Leuven, Belgium (CF, MvdV), the Department of Surgery, DIAKO Ev. Diakonie-Krankenhaus Bremen, Germany (SMF), the Department of Anaesthesiology and Pain Management, University of Texas Southwestern Medical Center, Dallas, Texas, USA (GPJ)

## Abstract

**BACKGROUND:**

Haemorrhoidectomy is associated with moderate-to-severe postoperative pain.

**OBJECTIVE:**

The aim of this systematic review was to assess the available literature and update previous PROSPECT (procedure specific postoperative pain management) recommendations for optimal pain management after haemorrhoidectomy.

**DESIGN AND ELIGIBILITY CRITERIA:**

A systematic review utilising PROSPECT methodology was undertaken.

**DATA SOURCES:**

Randomised controlled trials published in the English language from January 1, 2016 to February 2, 2022 assessing postoperative pain using analgesic, anaesthetic, and surgical interventions were identified from MEDLINE, EMBASE and Cochrane Database.

**RESULTS:**

Of the 371 randomized controlled trials (RCTs) identified, 84 RCTs and 19 systematic reviews, meta-analyses met our inclusion criteria (103 publications). Interventions that improved postoperative pain relief included: paracetamol and nonsteroidal anti-inflammatory drugs or cyclo-oxygenase-2 selective inhibitors, systemic steroids, pudendal nerve block, topical metronidazole, topical diltiazem, topical sucralfate or topical glyceryl trinitrate, and intramuscular injection of botulinum toxin.

**DISCUSSION:**

This review has updated the previous recommendations written by our group. Important changes are abandoning oral metronidazole and recommending topical metronidazole, topical diltiazem, topical sucralfate, topical glyceryl trinitrate. Botulinum toxin can also be administered. Contemporary publications confirm the analgesic effect of bilateral pudendal nerve block but invalidate recommendations on perianal infiltration. The choice of the surgery is mostly left to the discretion of the surgeons based on their experience, expertise, type of haemorrhoids, and risk of relapse. That said, excisional surgery is more painful than other procedures.

## Introduction

Pain after haemorrhoid surgery is moderate to severe. Multiple pharmacological treatments, anaesthetic strategies and surgical techniques have been investigated to provide postoperative analgesia. Previous PROSPECT (procedure-specific pain management) guidelines for pain management have been published in 2010^1^ and 2017.^2^ The PROSPECT Working Group is a collaboration of anaesthesiologists and surgeons working to formulate procedure specific recommendations for pain management after common surgical procedures. The recommendations are based on a procedure-specific literature review of randomised controlled trials (RCTs) and systematic reviews. A special feature of PROSPECT recommendations is that the methodology considers clinical practice, efficacy, and adverse effects of analgesic techniques.^3^

As many trials have been published since our last recommendations on haemorrhoid surgery, we decided to provide an update. The objective of this review was to systematically assess the available literature on pain management after any haemorrhoid surgery. Postoperative pain outcomes (pain scores and analgesic requirements) were the primary outcomes. Other recovery outcomes, including adverse effects, were also evaluated, and the limitations of the data were reviewed.

## Methods

We adhered to previously described PROSPECT methodology in the conduct of this project.^3^ This systematic review was registered on PROSPERO (CRD42022323482, registered in May, 2022). For this study, we specifically searched the following databases from January 1, 2016 to February 2, 2022 for any randomised controlled trials investigating any intervention for haemorrhoid surgery and reporting pain scores: the US National Library of Medicine Database (MEDLINE), the Excerpta Medica database (EMBASE), and the Cochrane Central Register of Controlled Clinical Trials.

The intervention search terms applied, and keywords were, among others: haemorrhoidectomy, Milligan-Morgan, excision, perioperative, and postoperative. Deduplication of the retrieved records was done manually. Population limits were then applied including clinical trials OR random allocation OR therapeutic use. Details of this literature search are provided in Appendix 1, Supplemental Digital Content.

We excluded any article describing a phase II study for a drug that was unlicensed at the time of this review, and any study that compared different agents, dosages, concentrations or analgesic techniques with no control group.

Quality assessment, data extraction and data analysis adhered to the PROSPECT methodology.^3^ Pain intensity scores were used as the primary outcome measure. We defined a change of more than 1 unit on a numerical rating score (NRS) as clinically relevant: if a visual analogue scale (VAS) was used we defined a unit as 10 mm. The effectiveness of each intervention for each outcome was evaluated by assessing the differences reported between treatment arms in each study. A meta-analysis was not performed due to heterogeneity in study design and result reporting restricting pooled analysis. Recommendations were made according to PROSPECT methodology.^3^ The proposed recommendations were sent to the PROSPECT Working Group for review and comments and a modified Delphi approach was utilised as previously described. Once a consensus was achieved the lead authors drafted the final document, which was ultimately approved by the Working Group.

## Results

**FIGURE 1:**
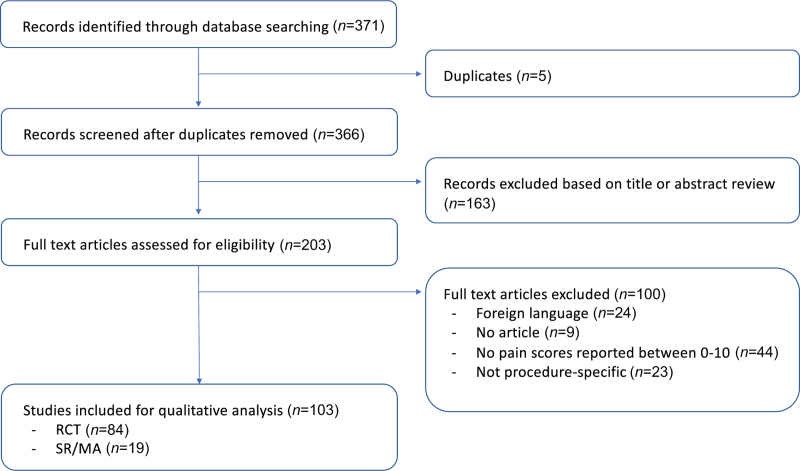
PRISMA flow diagram of studies.

Among the 371 articles retrieved from the literature search and one article from bibliography screenings, 84 RCTs^4–87^ and 19 systematic reviews^88–106^ were finally included (total: 103 publications, Fig. 1). Table S1, Supplemental Digital Content provides a summary of key results of studies used to support the recommended interventions, while Table S2, Supplemental Digital Content provides a summary of key results of studies assessing interventions that were not recommended. Table S3, Supplemental Digital Content lists the studies excluded and the reasons for exclusion.

### Pharmacological treatments

A meta-analysis on 5 trials, 337 patients demonstrated that oral metronidazole did not reduce postoperative pain from day 0 to day 7, except on day 4.^102^ The absence of analgesic effect was confirmed in a subsequent trial on 40 patients followed for 21 days where basic analgesics were prescribed.^77^ On the other hand, two meta-analyses on 8 trials, 437 patients,^97^ and 9 trials, 523 patients^103^ pooling data from trials investigating oral or topical metronidazole identified the superiority of metronidazole in reducing pain scores with mean differences of 1.2^103^ and 1.4 units^97^ on postoperative day 1 and 2.2^103^ and 2.4 units^97^ on postoperative day 7. A meta-analysis pooled two trials comparing topical metronidazole with a control group and demonstrated a reduction in pain score by more than one unit up to postoperative day 14.^104^ Three trials compared oral versus topical metronidazole.^4,55,80^ Two of these (166 patients^4^ and 120 patients^55^) showed a significant reduction in pain scores by more than one unit up to postoperative day 7, without specifying whether basic analgesics were prescribed or not. The third trial on 120 patients did not find any difference between groups but used a wide postoperative analgesic regimen inclusive of paracetamol, ibuprofen, wound infiltration and bilateral pudendal nerve block.^80^

A systematic review and meta-analysis on topical treatments included 32 trials and concluded that topical diltiazem and topical sucralfate reduced postoperative pain scores postoperatively by more than 1.5 units.^104^ Regarding topical diltiazem, these results were confirmed with a control group in subsequent trials with 227,^93^ 60,^82^ 58,^14^ and 80^5^ patients. Administration of basic analgesics was reported in one study.^82^ Regarding topical sucralfate, Vejdan *et al.* endorsed the findings in a trial of 40 patients, but without prescribing basic analgesics.^72^ A meta-analysis on topical glyceryl trinitrate included 12 trials (1095 patients) and demonstrated a reduction in pain score by a mean difference of 1.2 units that persisted up to postoperative day 14.^95^ These results were confirmed by a subsequent trial on 40 patients.^71^

The following topical treatments showed reductions in postoperative pain scores in single studies when compared with a control group: atorvastatin gel 2% for 14 days (*n* = 66),^7^ baclofen for 14 days (*n* = 66).^8^ In a cohort of 120 patients, topical lidocaine 2% combined with diclofenac provides more postoperative analgesia than topical lidocaine 2%.^44^ None of these three studies used routine basic analgesics.

Rabelo *et al.* allocated 68 patients in 4 arms: placebo, oral metronidazole, oral flavonoids, oral flavonoids combined with oral metronidazole.^54^ The authors showed a reduction in pain scores in patients receiving flavonoids by a mean difference of 3 units on postoperative day 7, while there was no outcome difference between patients receiving oral metronidazole alone versus placebo.^54^

Two trials with 67^12^ and 82 patients^65^ investigated the analgesic efficacy of botulinum toxin injection during the procedure, while prescribing basic analgesics, and demonstrated a reduction of more than one unit in pain scores up to postoperative day 14. The injection is more effective when performed one week before the surgery as demonstrated by Cheng *et al.* in a trial of 62 patients.^20^ No side-effects such as anal incontinence were reported.

Yeh *et al.* investigated the analgesic benefit of sebacoyl dinalbuphine ester injected intramuscularly prior to surgery in a total of 221 patients and demonstrated a reduction in pain score by 0.4 units on postoperative day 2;^83^ no basic analgesics were prescribed.

### Anaesthetic and analgesic strategies

Nine publications examined the analgesic efficacy of a bilateral pudendal nerve block.^24,28,31,38,49,52,66,94,98^ One meta-analysis of 7 trials including a total of 560 patients concluded that, compared to a control group, the bilateral block versus a reduced pain score by a mean difference of 1.9 units at 12 h and 0.5 units at 24 h.^94^ Another meta-analysis on 14 trials which included a total of 1214 patients concluded that the bilateral pudendal nerve block provides superior analgesia when compared with perianal infiltration or a control group by mean differences in pain scores of 3.1 and 2.1 units at 12 and 24 postoperative hours, respectively.^98^ The superiority of pudendal nerve block over a control group was recently confirmed by a prospective trial on 49 patients that prescribed basic analgesics;^24^ indeed, Di Giuseppe *et al.* showed a reduction in pain scores of 1.8 and 1.7 units at 6 and 24 postoperative hours.^24^ Notably pudendal nerve blocks do not provide additional analgesia when combined with perianal infiltration and compared with perianal infiltration alone^66^ or when compared with an intravenous patient-controlled analgesia of morphine.^31^ When compared with a spinal anaesthetic, four publications showed concordant results;^28,38,49,52^ while Perivoliotis *et al.* (*n* = 60) and Nadri *et al.* (*n* = 70) identified a reduction in pain scores at 12 and 24 h by 3.8^52^ and 0.8 units,^49^ respectively, Kumar *et al.* (*n* = 50) showed no difference on postoperative day 1 and 2 when all patients received a 50 mg pethidine intramuscular injection.^38^ Finally, He *et al.* (*n* = 118) compared a bilateral pudendal nerve block with ropivacaine combined with dexmedetomidine and a target controlled infusion of propofol combined with sufentanil to spinal anaesthesia with ropivacaine and an intravenous infusion of dexmedetomidine. They demonstrated the superiority of the first group at 12 and 24 postoperative hours by mean differences of 1.9 and 2.0 units, respectively.^28^

Regarding spinal anaesthesia, Borges *et al.* (*n* = 40) compared this technique to perianal infiltration combined with general anaesthesia and showed a 2.5-unit reduction in pain scores up to 2 postoperative hours in patients allocated to spinal anaesthesia, while basic analgesics were prescribed.^17^ If 50 μg morphine is added intrathecally, analgesia is prolonged up to 24 postoperative hours with a mean reduction in pain scores of 1.8 units, as demonstrated in a prospective trial of 66 patients.^57^

Four studies investigated local anaesthetic wound infiltration^13,22,27,36^ but only one included basic analgesics.^13^ The authors compared wound infiltration with bupivacaine (*n* = 90),^27^ ropivacaine (*n* = 50),^13^ lidocaine (*n* = 111)^22^ and liposome bupivacaine (*n* = 92)^36^ versus a control group and none demonstrated a reduction in pain scores. Notably one of these studies also included an arm with tramadol infiltration and showed a mean 0.7 unit reduction in pain score during the first 24 h when compared with a control group;^27^ no basic analgesics were prescribed. Finally, the study investigating liposome bupivacaine also included an arm of patients receiving a mixture of liposome bupivacaine with aloe vera and demonstrated a mean 1.5-unit reduction in pain score when compared with the control group up to 36 postoperative hours;^36^ again, no basic analgesics were prescribed.

### Surgical procedures

Fifty-four studies investigated different surgical techniques, such as Milligan–Morgan, Ferguson, stapled haemorrhoidopexy, LigaSure haemorrhoidectomy, artery ligation or ultrasonic procedures.^6,9–11,15,16,18,19,25,26,30,32,34,35,37,39–43,45,47,48,50–53,56,58,60–62,64,67–70,73,74,76,78,81,85,86,88–92,96,100,101,105,106^

Three trials with 777 patients,^76^ 258,^58^ and 244 patients,^37^ consistently concluded that stapled haemorrhoidopexy reduced postoperative pain when compared with excisional surgery. This conclusion was corroborated by a systematic review on 38 studies.^100^ Lin *et al.* concluded after including 244 patients that partial stapled haemorrhoidopexy reduced pain scores up to postoperative day 7 when compared with a circumferential technique, while Hidalgo-Grau *et al*. did not find any difference between groups in 119 patients having a fixation at 4.5 or 6 cm of the external anal verge.^30^ Stapled haemorrhoidopexy also reduced postoperative pain in a trial of 110 patients when compared with excisional surgery.^47^ A systematic review on 5 trials, 262 patients comparing stapled with LigaSure haemorrhoidopexy did not find any difference between the groups.^106^

Five trials comparing LigaSure with Milligan–Morgan haemorrhoidectomy on 130,^9^ 66,^62^ 55,^15^ 60,^11^ and 240 patients^68^ concluded that LigaSure produces less postoperative pain. LigaSure was also less painful when compared with radiofrequency haemorrhoidectomy in a trial of 50 patients.^45^

Five trials compared ultrasonic techniques with excisional haemorrhoidectomy on 50,^42^ 160,^61^ 240,^68^ 50,^10^ and 130 patients.^64^ Except for one trial,^64^ they all demonstrated a reduction in pain scores with the ultrasonic technique. Of note, one of these studies included an arm with LigaSure and demonstrated similar postoperative analgesia when compared with ultrasonic technique.^68^

A systematic review of 14 trials including a total of 1570 patients concluded that nonexcisional laser therapies produce less pain than haemorrhoidectomy or rubber band ligation.^96^ This publication included two trials captured by our literature search.^48,53^ We captured an additional study on 80 patients showing no difference between groups.^60^

Contrasting results are reported with transanal haemorrhoidal de-arterialisation when compared with stapled haemorrhoidectomy. While a meta-analysis on 9 trials including a total of 1077 patients^105^ reported similar pain postoperatively, another meta-analysis on 6 trials with a total of 554 patients demonstrated a pain reduction by a mean difference of 0.4 units.^92^ Two studies included in one of these meta-analyses^105^ showed no group difference^73^ or a reduction in pain by only 0.6 units.^40^ Two subsequent trials on 89^56^ and 40 patients^19^ favoured transanal haemorrhoidal de-arterialisation. When compared with LigsaSure excision (*n* = 80)^39^ or the ultrasonic technique (*n* = 44),^70^ transanal haemorrhoidal de-arterialisation produces less pain, with a mean differences of 0.7^70^ and 2.9 units^39^ at 24 postoperative hours. Compared to mucopexy,^6,85^ vessel-sealing device haemorrhoidectomy^69^ and tissue selective technique,^41^ transanal haemorrhoidal de-arterialisation produces similar pain, while injection of aluminium potassium sulfate and tannic acid combined with mucopexy produces less pain.^67^

Rubber band ligation produces less pain postoperatively than excisional haemorrhoidectomy in a meta- analysis on 8 trials with a total of 1208 patients^91^ and in a trial of 120 patients.^35^ Rubber band ligation was also superior to arterial ligation (*n* = 372)^18^ but inferior to haemorrhoid energy therapy (*n* = 30)^26^ and suction (*n* = 60).^16^

Two recent network meta-analyses, one including 26 trials with a total of 3137 patients^88^ and the other 29 trials with 3309 patients,^89^ concluded that excisional haemorrhoidectomy is associated with more pain, while stapled, laser, or ultrasonic techniques were associated with less pain. A meta-analysis on 11 trials and 1326 patients concluded that Ferguson haemorrhoidectomy reduces postoperative pain by a mean difference of 0.4 units when compared with Milligan-Morgan haemorrhoidectomy.^90^ When compared with infrared photocoagulation (*n* = 40)^51^ or electrotherapy (*n* = 120),^50^ Ferguson haemorrhoidectomy was associated with more pain. Milligan-Morgan haemorrhoidectomy produces less pain when associated with lateral internal sphincterotomy^74,101^ or purse string suture,^25^ and similar pain as submucosal haemorrhoidectomy^81^ or tissue selecting technique.^32^ On the other hand, Milligan–Morgan haemorrhoidectomy was more painful than segmental resection,^78^ high suspension technique^34^ or suture-fixation technique.^86^

### Other modalities

A trial compared routine care with an Enhanced Recovery after Surgery (ERAS) programme inclusive of preemptive pain control with paracetamol and gabapentin, nutrition optimisation, targeted education on constipation prevention, functional activity, and postoperative precautions on pain management, along with intraoperative multimodal opioid-sparing pain management with intravenous ketamine, intravenous dexamethasone and perianal infiltration.^23^ After including a total of 64 patients, the authors did not find any difference in pain scores during the first 30 postoperative days, while opioid consumption was globally reduced in the ERAS group.^23^ More specifically, a postoperative medication checklist did not lead to a reduction in pain scores up to 14 postoperative days in a trial on 35 patients who received basic analgesics.^33^

A network meta-analysis on 107 trials with a total of 10 972 patients concluded that acupuncture reduced pain scores postoperatively.^99^ This finding was confirmed by three subsequent trials on 144,^101^ 72^79^ and 80 patients^84^ with mean differences between 0.4^79^ and 0.8 units^75^ at 24 postoperative hours. Among these studies, only one reported the prescription of basic analgesics.^84^

Studies investigating the combined application of Shuangjin ointment with beta-sodium aescinate versus a control group in 150 patients,^87^ the modified Buzhong Yiqi decoction combined with Gangtai ointment versus chitosan hydrogels in 120 patients,^46^ or a sitz bath with Xiaozhi versus sitz bath with warm water in 310 patients^63^ showed reduction in pain score by less than one unit postoperatively in the intervention groups; none of these studies reported prescription of basic analgesics.

A trial on 60 patients comparing Karamardadi yoga with sodium diclofenac did not find any significant difference in analgesic outcomes between groups up to postoperative day 3.^29^ One study (*n* = 182) examined the analgesic effect of oral Venoplant^59^ administered for 30 consecutive days in the postoperative period without basic analgesics and showed a reduction in pain scores by less than one unit on postoperative day 14 and 30, but not on postoperative day 7.^59^ Finally, Chiaretti *et al.* included 94 patients and did not find any difference between patients receiving oral flavonoids and oral Centella Complex.^21^

## Discussion

After reviewing 103 articles published since 2016 and following the PROSPECT approach, we have updated our recommendations for analgesia following haemorrhoid surgery that are listed in Table 1. Table 2 summarises the analgesic interventions that are not recommended for pain management. Table 3 presents the evolution of the recommendations between this update and the two previous reviews.

**Table 1:** Overall recommendations for pain management in patients undergoing haemorrhoid surgery.

Pharmacological treatment
• Paracetamol combined with nonsteroidal anti-inflammatory drugs or cyclooxygenase (COX)-2 selective inhibitors administered preoperatively or intraoperatively and continued postoperatively
• Dexamethasone (intravenous, single dose)
• Laxatives
• Topical metronidazole, diltiazem, sucralfate or glyceryl trinitrate
• Botulinum toxin
• Opioid for rescue
Anaesthetic and analgesic strategies
• Bilateral pudendal nerve block
Surgical procedures
• The surgical technique should be left to the type of hemorrhoids and surgeon's experience and expertise. Of note, Milligan-Morgan haemorrhoidectomy is more painful than other surgical techniques.
Other modalities
• Acupuncture

**Table 2:** Analgesic interventions that are not recommended for pain management in patients undergoing haemorrhoid surgery.

	Intervention	Reason for not recommending
*Pharmacological treatments*	Oral metronidazole	Conflicting procedure-specific evidence
	Intramuscular sebacoyl dinalbuphine ester	Limited procedure-specific evidence
	Topical atorvastatin	Limited procedure-specific evidence
	Topical baclofen	Limited procedure-specific evidence
	Topical lidocaine with diclofenac	Limited procedure-specific evidence
*Anaesthetic and analgesic strategies*	Spinal anaesthesia	Limited procedure-specific evidence
	Intrathecal hydrophilic opioid	Limited procedure-specific evidence
	Perianal infiltration with tramadol	Limited procedure-specific evidence
	Perianal infiltration with plain local anaesthetic	Lack of procedure-specific evidence
	Perianal infiltration with liposome bupivacaine	Lack of procedure-specific evidence
	Perianal infiltration with liposome bupivacaine combined with aloe vera	Limited procedure-specific evidence
*Surgical procedures*	Milligan-Morgan haemorrhoidectomy	Lack of procedure-specific evidence
	Ferguson haemorrhoidectomy	Conflicting procedure-specific evidence
	Injection of aluminium potassium sulfate and tannic acid combined with mucopexy	Conflicting procedure-specific evidence
*Other modalities*	Postoperative medication checklist	Lack of procedure-specific evidence
	Topical Shuangjin ointment with beta- sodium aescinate	Limited procedure-specific evidence
	Modified Buzhong Yiqi decoction combined with Gangtai ointment	Limited procedure-specific evidence
	Sitz bath with Xiaozhi	Limited procedure-specific evidence
	Karamardadi yoga with sodium diclofenac	Lack of procedure-specific evidence
	Oral Venoplant	Limited procedure-specific evidence
	Oral flavonoids with Centella Complex	Lack of procedure-specific evidence

**Table 3:** Comparisons of the recommended interventions for pain management in patients undergoing haemorrhoid surgery with the previous PROSPECT recommendations.

	Joshi *et al.* 2010	Sammour *et al.* 2017	Bikfalvi *et al.* 2023
*Pharmacological treatment*			
Paracetamol	x	x	x
NSAIDs/COX-2 specific inhibitors	x	x	x
Parenteral glucocorticoids	x	x	x
Laxatives	x	x	x
Oral metronidazole	x	x	
Topical metronidazole		x	x
Topical diltiazem			x
Topical sucralfate			x
Topical glyceryl trinitrate		x	x
Botulinum toxin			x
*Anaesthetic and analgesic strategies*			
Perianal infiltration	x		
Biltateral pudendal nerve block	x	x	x
*Other modalities*			
Acupuncture			x

COX, cyclooxygenase; NSAID, nonsteroidal anti-inflammatory drug.

Pharmacological treatments for analgesia include paracetamol, nonsteroidal anti-inflammatory drugs (NSAIDs) or cyclooxygenase (COX)-2 specific inhibitors, started preoperatively and continued in the postoperative period, along with single dose of systemic steroids administered intraoperatively. Of note, no additional studies investigating these drugs have been published since 2010.^1^ Regarding steroids, two trials included previously investigated intramuscular betamethasone; however, dexamethasone is commonly used as an antiemetic and a simple increase in the dose to 0.1–0.2 mg kg^−1^ will provide extra analgesia.^107^ Due to concerns about opioid-related adverse effects such as postoperative nausea and vomiting,^108^ constipation with subsequent pain on defecation, and their contribution to the current opioid crisis^109^ opioids should only be considered as rescue analgesics, if the recommended approaches are not adequate.

Oral metronidazole was previously recommended; however, in the light of more recent publications that failed to demonstrate an analgesic effect, especially in the setting of multimodal analgesia, and the risk of antibiotic resistance, we cannot maintain this recommendation due to conflicting results. Oral metronidazole may still be used in routine clinical practice for reasons others than pain. In contrast, however, topical metronidazole does provide effective analgesia, so does topical diltiazem, topical sucralfate, and topical glyceryl trinitrate. In the absence of evidence, the choice of postoperative topical treatment should be left to the preferences of the surgeon and tailored to the individual characteristics of the patient. New evidence indicates that local injection of botulinum toxin reduces postoperative pain, but anal incontinence was not an outcome mentioned in the studies included. However, the cost of this intervention may be prohibitive in routine practice.

While perianal infiltration was recommended initially,^1^ we confirm that this analgesic technique could be abandoned due to the lack of evidence in a contemporary practice. Concordant evidence continues to point towards the analgesic benefit of the bilateral pudendal nerve block, also called ischiorectal block, explaining why this recommendation is maintained. However, the risk of nerve injury and the subsequent pudendal neuralgia, especially if the block is performed blindly, should be balanced against the analgesic benefit. Perioperative acupuncture can be recommended as an analgesic adjunct. However, heterogeneity in the techniques of the different articles included, along with a pain score reduction of less than one unit, and the required specific training preclude wide dissemination in clinical practice. Finally, an ERAS program does not reduce pain scores *per se*, probably due to the multiple factors included in an ERAS program. But as it reduces opioid consumption in the postoperative period, we included this item in our recommendations.

Regarding the surgical techniques, a myriad of different procedures has been developed and compared between themselves, making the establishment of firm recommendations difficult. The surgical technique should be left to the type of haemorrhoids and the surgeon's experience and expertise. That said, Milligan-Morgan haemorrhoidectomy is more painful than stapled haemorrhoidopexy, LigaSure haemorrhoidectomy and ultrasonic procedures, which in turn are more painful than transanal haemorrhoidal de-arterialisation or rubber band ligation.

The limitations in this review are related to those of the included studies. There was considerable heterogeneity between studies with regards to dosing regimens and surgical techniques, as well as timing of pain assessments. The small size of many studies has the potential for estimation effect and do not provide safety profiles for the analgesic interventions. In most of the studies, the analgesic intervention was not evaluated against an optimised multimodal analgesic regimen. Indeed, in many of the trials, the patients did not receive basic analgesics including paracetamol or nonsteroidal anti-inflammatory drugs. For example, no trial investigated the analgesic benefit of a topical preparation in addition to routine administration of paracetamol and a nonsteroidal anti-inflammatory drug.

In conclusion, this review has updated the previous recommendations written by our group. Important changes relate to abandoning oral metronidazole and recommending topical metronidazole, topical diltiazem, topical sucralfate, and topical glyceryl trinitrate. Botulinum toxin can also be administered. Contemporary publications confirm the analgesic effect of bilateral pudendal nerve blocks but invalidate recommendations on perianal infiltration. The choice of the surgery is mostly left to the discretion of the surgeon based on experience, expertise, type of haemorrhoids, and risk of relapse. That said, excisional surgery is more painful than other procedures. Due to the wide heterogeneity of the surgical procedures, proper randomised controlled trials should evaluate more systematically and rigorously the analgesic benefit of different interventions.

## Supplementary Material

Supplemental Digital Content

## Supplementary Material

Supplemental Digital Content

## Supplementary Material

Supplemental Digital Content

## Supplementary Material

Supplemental Digital Content
