# Supplementary material for: PROSPECT guideline for haemorrhoid surgery: A systematic review and procedure-specific postoperative pain management recommendations
Source: Eur J Anaesthesiol Intensive Care. 2023 May 26;2(3):e0023. doi: 10.1097/EA9.0000000000000023 (PMC11783633; doi:10.1097/EA9.0000000000000023)
Supplement: Supplemental Digital Content [file ejaic-2-e0023-s001.docx]

Appendix: **Search strategies**

| Databases | Date | Number of references | |
| --- | --- | --- | --- |
|  |  | found | afterdeduplication |
| Medline ALL Ovid  1946 to February 15, 2022 | 16-Feb-22 | 631 | 630 |
| Embase.com | 16-Feb-22 | 842 | 262 |
| Cochrane Central Register of Controlled Trials Wiley  Issue 2 of 12, February 2022 | 16-Feb-22 | 528 | 36 |
| Total |  | 2001 | 928 |

**Embase.com**

842 references on 16.02.2022

('hemorrhoidectomy'/de OR 'hemorrhoid'/exp/dm_su OR ('hemorrhoid'/exp AND 'vascular surgery'/exp) OR ('hemorrhoid'/exp/mj AND 'surgery'/exp) OR (haemorrhoidectom* OR hemorrhoidectom* OR haemorrhoidopex* OR hemorrhoidopex* OR posthaemorrhoidectom* OR posthemorrhoidectom* OR Haemorrhoidoplast* OR Hemorrhoidoplast* OR ((hemorrhoid* OR haemorrhoid* OR piles) NEAR/12 (surgery OR surgical* OR operat* OR preoperative* OR postoperative* OR perioperative* OR excision* OR Milligan-Morgan OR Ferguson OR parks OR "suture fixation" OR laser OR staple* OR scalpel OR longo OR electrocoagulation OR coagulation OR electrocauter* OR cauter* OR diatherm* OR electrothermal OR ligation OR ligasure OR Surgitron))):ab,ti,kw) AND ('Controlled clinical trial'/exp OR 'Crossover procedure'/de OR 'Double-blind procedure'/de OR 'Single-blind procedure'/de OR (random* OR factorial* OR crossover* OR (cross NEXT/1 over*) OR placebo* OR ((doubl* OR singl*) NEXT/1 blind*) OR assign* OR allocat* OR volunteer* OR trial OR groups):ab,ti,kw) NOT ([animals]/lim NOT [humans]/lim) NOT ('conference abstract'/it OR 'conference review'/it) AND [2006-2022]/py

Filter applied for randomized control trials adapted from Cochrane Handbook, Version 5.0.1, [updated March 2011], eds: Higgins & Green

**Medline ALL Ovid**

1946 to February 15, 2022

631 references on 16.02.2022

(Hemorrhoidectomy/ OR Hemorrhoids/su OR (Hemorrhoids/ AND exp Vascular Surgical Procedures/) OR (*Hemorrhoids/ AND exp Surgical Procedures, Operative/) OR (haemorrhoidectom* OR hemorrhoidectom* OR haemorrhoidopex* OR hemorrhoidopex* OR posthaemorrhoidectom* OR posthemorrhoidectom* OR Haemorrhoidoplast* OR Hemorrhoidoplast* OR ((hemorrhoid* OR haemorrhoid* OR piles) ADJ12 (surgery OR surgical* OR operat* OR preoperative* OR postoperative* OR perioperative* OR excision* OR Milligan-Morgan OR Ferguson OR parks OR "suture fixation" OR laser OR staple* OR scalpel OR longo OR electrocoagulation OR coagulation OR electrocauter* OR cauter* OR diatherm* OR electrothermal OR ligation OR ligasure OR Surgitron))).ab,ti,kf.) AND (exp Controlled clinical trial/ OR "Double-Blind Method"/ OR "Single-Blind Method"/ OR "Random Allocation"/ OR (random* OR factorial* OR crossover* OR cross over* OR placebo* OR ((doubl* OR singl*) ADJ blind*) OR assign* OR allocat* OR volunteer* OR trial OR groups).ab,ti,kf.) NOT (exp Animals/ NOT Humans/)

Limit search to yr="2006 -Current"

Filter applied for randomized control trials adapted from Cochrane Handbook, Version 5.0.1, [updated March 2011], eds: Higgins & Green

**Cochrane Central Register of Controlled Trials Wiley**

Issue 2 of 12, February 2022

528 references on 16.02.2022

(haemorrhoidectom* OR hemorrhoidectom* OR haemorrhoidopex* OR hemorrhoidopex* OR posthaemorrhoidectom* OR posthemorrhoidectom* OR Haemorrhoidoplast* OR Hemorrhoidoplast* OR ((hemorrhoid* OR haemorrhoid* OR piles) NEAR/12 (surgery OR surgical* OR operat* OR preoperative* OR postoperative* OR perioperative* OR excision* OR "Milligan Morgan" OR Ferguson OR parks OR "suture fixation" OR laser OR staple* OR scalpel OR longo OR electrocoagulation OR coagulation OR electrocauter* OR cauter* OR diatherm* OR electrothermal OR ligation OR ligasure OR Surgitron))):ab,ti,kw

Limits: 2006-2022

- Exclusion of conference abstracts and clinical trial registry references
