# Supplementary material for: PROSPECT guideline for haemorrhoid surgery: A systematic review and procedure-specific postoperative pain management recommendations
Source: Eur J Anaesthesiol Intensive Care. 2023 May 26;2(3):e0023. doi: 10.1097/EA9.0000000000000023 (PMC11783633; doi:10.1097/EA9.0000000000000023)
Supplement: Supplemental Digital Content [file ejaic-2-e0023-s002.docx]

**Table S1.** Summary of key results from studies evaluating pharmacological treatments, anaesthetic analgesic strategies, surgical procedures, and other modalities used to support the recommended interventions in patients after haemorrhoid surgery. Bid, *bis in die*, twice a day; MA, meta-analysis; NS, not significant; NSAID, non-steroidal anti-inflammatory drugs; POD, postoperative day; SR, systematic review; tid, *ter in die,* three times a day**.**

| **Study** | **Study design** | **Pain scores** | **Cumulative opioid consumption** | **Basic analgesia administered** |
| --- | --- | --- | --- | --- |
| **PHARMACOLOGICAL TREATMENTS** | | | | |
| ***Topical metronidazole*** | | | | |
| Xia et al. 2020^104^ | Topical metronidazole vs control (MA, 2 trials, 201 patients) | Favours metronidazole on POD1 (mean difference of 1.1; *P*=0.01) and POD14 (*P*<0.01) | Not reported | None |
| ***Topical versus oral metronidazole*** | | | | |
| Abbas et al. 2020^4^ | Topical metronidazole 10% tid (*n*=83) vs oral metronidazole 400mg tid (*n*=83) | Favours topical metronidazole on POD7 (mean difference of 1.0; *P*<0.01) | Not reported | Not reported |
| Razzaq et al. 2020^55^ | Topical metronidazole (*n*=60) vs oral metronidazole (dosages not reported)(*n*=60) | Favours topical metronidazole on POD3 (mean difference of 1.2; *P*=0.02) and on POD5 (*P*=0.01) | Not reported | Not reported |
| Xia et al. 2021^80^ | Topical metronidazole 10% tid (*n*=60) vs oral metronidazole 400mg tid (*n*=60) | NS on POD1 to POD14 | NS | Acetaminophen ibuprofen, bilateral pudendal nerve block, perianal infiltration, tramadol as needed |
| ***Topical calcium channel blockers*** | | | | |
| Xia et al. 2020^104^ | Topical diltiazem vs control (MA; 5 trials; 530 patients) | Favours diltiazem on POD1 (mean difference of 2.9; *P*<0.01) and POD7 (p<0.01). | Not reported | None |
| Huang et al. 2018^93^ | Topical diltiazem (*n*=137) vs control (*n*=90) (MA, 5 trials, 227 patients) | Favours diltiazem on POD2 (mean difference of 3.4), POD3 (*P*<0.01) and POD4 (*P*<0.01) | Not reported | Not reported |
| Yadav et al. 2018^82^ | Topical diltiazem 2% (*n*=30) vs control (*n*=30) | Favours topical calcium channel blockers on POD1 (mean difference of 2.1) and 7 (*P*<0.01) | Not reported | Diclofenac |
| Bader et al. 2020^14^ | Topical gel with diltiazem 2% and diclofenac 1%, lidocaine 2% (*n*=29) vs topical gel with lidocaine 2% and diclofenac 1% (*n*=29) | Favours diltiazem, diclofenac, lidocaine on POD3 (mean difference of 4.7; *P*=0.03) and POD6 (*P*=0.01) | Not reported | Not reported |
| Abidi et al. 2021 ^5^ | Topical diltiazem 2% (*n*=40) vs control (*n*=40) | Favours topical diltiazem on POD1 (mean difference of 1.8; *P*<0.01) and POD3 (mean difference of 1.9; *P*<0.01) | Not reported | Not reported |
| ***Topical sucralfate*** |  |  |  |  |
| Vejdan et al. 2020^72^ | Topical 10% sucralfate (*n*=20) vs control (*n*=20) | Favours sucralfate on POD13 (mean difference of 3.2; *P*<0.01) | Not reported | Not reported |
| Xia et al. 2020^104^ | Topical sucralfate vs control (MA, 2 trials, 328 patients) | Favours sucralfate on POD7 (mean difference of 1.7; *P*<0.01) and 14 (*P*<0.01) | Not reported | None |
| ***Topical glyceryl trinitrate*** | | | | |
| Xia et al. 2020^104^ | Topical glyceryl trinitrate vs control (MA, 9 trials, 1230 patients) | Favours glyceryl trinitrate on POD3 (mean difference of 1.7; *P*=0.04) and POD7 (*P*=0.02). | Not reported | None |
| Liu et al.2016^95^ | Effect of topical glyceryl trinitrate on pain control (MA, 12 trials, 1095 patients) | Favours glyceryl trinitrate on POD1 (mean difference of 1.2; *P*=0.04), up to POD14 (*P*<0.01) | Not reported | Not reported |
| Vahabi et al. 2018^71^ | Glyceryl trinitrate ointment 0.2% (*n*=20) vs control (*n*=20) | Favours glyceryl trinitrate at 12h, 18h and 24h (mean difference of 4.2; *P*<0.01) | Not reported | Meperidine as needed |
| ***Botulinum toxin*** |  |  |  |  |
| Alvandipour. 2021^12^ | Botulinum toxin injection, 20 U (*n*=34) vs control) (*n*=33) | Favours botulinum toxin at 12h and on POD1 (mean difference of VAS of 1.5; *P*<0.01), POD2, POD7 and POD14 (*P*<0.01) | Favours botulinum toxin for the first 24h (*P*<0.01) | Acetaminophen, morphine as needed |
| Sirikurnpiboon et al. 2020^65^ | Botulinum toxin injection, 30 U (*n*=39) vs control (*n*=43) | Favours botulinum toxin at 12h (mean difference of 1.8) and on POD1 (mean difference of 1.5; *P*<0.01) | NS | Acetaminophen, diclofenac, pethidine as needed |
| Cheng et al. 2022^20^ | Botulinum toxin injection, 50 U, injection one week before the operation (*n*=31) vs at the end of surgery (*n*=31) | Favours early injection on POD1 (mean difference of 1.5; *P*=0.01), POD2 (*P*=0.03), up to POD5 (*P*=0.01) | NS | Acetaminophen, flurbiprofen, tramadol, pethidine as needed |
| **ANAESTHETIC AND ANALGESIC STRATEGIES** | | | | |
| ***Bilateral pudendal nerve block*** | | | | |
| Li et al. 2021^94^ | Bilateral pudendal nerve block vs control (SR, 7 trials, 560 patients) | Favours bilateral pudendal nerve block at 6h (*P*<0.01), 12h (*P*<0.01) and 24h (mean difference of 0.5; *P*=0.04) | Favours bilateral pudendal nerve block (*P*<0.01) | None |
| Mongelli et al. 2021^98^ | Bilateral pudendal nerve block vs perianal infiltration, or control (MA, 14 trials, 1214 patients) | Favours bilateral pudendal nerve block at 6h (*P*<0.01), 12h (*P*<0.01), 24h (mean difference of 2.1; *P*<0.01) | Favours bilateral pudendal nerve block (*P*<0.01) | None |
| Di Giuseppe et al. 2020 ^24^ | Bilateral pudendal nerve block with ropivacaine 0.75%, 10 mL (*n*=23) vs control (*n*=26) | Favours bilateral pudendal nerve block at 6h (mean difference of 1.8; *P*<0.05) and 24h (mean difference of 1.7; *P*=0.02) | NS | Postoperative NSAID, opioid as needed) |
| Steen et al. 2020^66^ | Bilateral pudendal nerve blocks with bupivacaine 0.5% and adrenaline 5 µg.ml^-1^, 10 mL (*n*=39) vs control (*n*=40) | NS at 4h, 8h, 12h and 24h | NS | Acetaminophen, ibuprofen, local wound infiltration, oxycodone as needed |
| Honar et al. 2018^31^ | Bilateral pudendal nerve block with bupivacaine 0.5%, 5 mL (*n*=47) vs patient-controlled analgesia of morphine (*n*=42) | NS | Not reported | None |
| Nadri et al. 2018^49^ | Bilateral pudendal nerve block with bupivacaine 0.5%, 3 mL (*n*=35) vs spinal anaesthesia with bupivacaine 0.5%, 3 mL (*n*=35) | Favours bilateral pudendal nerve block on POD1 (mean difference of 0.8; *P*<0.05) | NS | Pethidine as needed |
| Perivoliotis et al. 2021^52^ | Bilateral pudendal nerve block with lidocaine, concentration not specified, 20 mL (*n*=30) vs spinal anaesthesia with levobupivacaine and fentanyl, concentrations not specified (*n*=30) | Favours bilateral pudendal nerve block at 12h (mean difference of 3.8; *P*<0.01) and on POD7 | Not reported | Acetaminophen, lornoxicam |
| Kumar et al. 2016^38^ | Bilateral pudendal nerve block with bupivacaine and lidocaine, unknown concentrations, 40 mL (*n*=25) vs spinal anaesthesia with bupivacaine 0.125%, 5 mL (*n*= 25) | NS on POD1 and 2 | Not reported | Pethidine |
| He et al. 2021^28^ | Bilateral pudendal nerve block with ropivacaine 0.4%, 30 mL, dexmedetomidine 0.5 µg.kg^-1^ along with intravenous propofol and sufentanil (*n*=58) vs spinal anaesthesia with hyperbaric ropivacaine 12.5 mg, along with intravenous dexmedetomidine 0.5 µg.kg.h^-1^ (n=60) | Favours bilateral pudendal nerve block at 3h, 6h, 12h, 24h (mean difference of 2.0; *P*<0.01) | Favours bilateral pudendal nerve block (*P*<0.001) | Acetaminophen, oxycodone, PCA of sufentanil |
| **SURGICAL PROCEDURES** | | | | |
| ***Stapled haemorrhoidopexy, haemorrhoidectomy*** | | | | |
| Watson et al. 2016^76^ | Traditional excisional surgery (*n*=388) vs stapled haemorrhoidopexy (*n*=389) | Favours stapled haemorrhoidopexy on POD7 and 21 (*P*<0.01) | Not reported | Not reported |
| Samee et al. 2018^58^ | Standard haemorrhoidectomy (*n*=129) vs stapled haemorrhoidopexy (*n*=129) | Favours stapled haemorroidopexy on (mean difference of 0.3; *P*<0.05) | Not reported | Not reported |
| Khan et al. 2020^37^ | Stapled haemorrhoidopexy (*n*=122) vs Milligan-Morgan haemorrhoidectomy (*n*=122) | Favours stapled haemorrhoidopexy (no timepoint, mean difference of 3.6; *P*<0.01) | Not reported | Not reported |
| Ruan et al. 2021^100^ | Milligan Morgan haemorrhoidectomy vs stapled haemorrhoidopexy (SR, 38 trials, number of patients unknown) | Favours stapled haemorrhoidopexy up to several postoperative days | Not reported | Not reported |
| Lin et al. 2019^43^ | Partial stapled haemorrhoidopexy (*n*=137) vs circumferential stapled haemorrhoidopexy (*n*=133) | Favours partial stapled haemorrhoidectomy on POD1 (mean difference 0f 0.2; *P*<0.01). | Not reported | Not reported |
| Hidalgo-Grau et al. 2020^30^ | Stapled anopexy at a distance of 4.5cm from external anal verge (*n*=58) vs 6cm (*n*=61) | NS on POD7 | Not reported | Acetaminophen, metamizole, diclofenac |
| Mir Mohammed et al. 2021^47^ | Conventional (*n*=55) vs stapled haemorrhoidectomy (*n*=55) | Favours stapled haemorrhoidectomy on POD2 (difference in VAS of 0.9; *P*=0.02) and POD3 (difference in VAS of 1.5; *P*<0.01), NS on POD1 | Favours stapled haemorrhoidectomy (*P*<0.01). | Not reported |
| Zhang et al. 2022^106^ | LigaSure® haemorrhoidectomy vs stapled (MA, 5 trials, 397 patients) | NS (no timepoint) | Not reported | Not reported |
| ***LigaSure® haemorrhoidectomy*** | | | | |
| Alhamdany et al. 2022^9^ | Milligan Morgan haemorrhoidectomy (*n*=50) vs LigaSure® haemorrhoidectomy (*n*=70) | Favours LigaSure® on POD5 (mean difference of 1.2; *P*<0.01) | Not reported | Not reported |
| Sharma et al. 2020^62^ | Milligan Morgan haemorrhoidectomy (*n*=33) vs LigaSure® haemorrhoidectomy (*n*=33) | Favours LigaSure® at 12h (mean difference of 0.6; *P*=0.01) and on POD7 (*P*=0.04) | Not reported | Diclofenac |
| Bakhtiar et al. 2016^15^ | Miligan-Morgan haemorrhoidectomy (*n*=26) vs LigaSure® (*n*=29) | Favours LigaSure® on POD1 (mean difference of 1.8; *P*<0.01) and 7 (*P*<0.01) | Not reported | Acetaminophen, ketorolac |
| Aljabery et al. 2020^11^ | Millian-Morgan haemorrhoidectomy (*n*=30) vs LigaSure® (*n*=30) | NS on POD1 and 7 | Not reported | Not reported |
| Talha et al. 2017^68^ | LigaSure® (*n*=60) vs conventional diathermy (*n*=60) vs Harmonic Scalpel (*n*=60) | Favours LigaSure® and Harmonic scalpel on POD1 up to POD7 (*P*<0.01). NS for LigaSure® vs Harmonic scalpel | Not reported | Diclofenac, oral metronidazole, glyceryl trinitrate cream |
| Lisi et al. 2019^45^ | Radiofrequency haemorrhoidectomy) (*n*=25) vs LigaSure® (*n*=25) | Favours LigaSure® on discharge and after first bowel movement (mean difference of 2.0; *P*<0.05) | Not reported | Acetaminophen, ketorolac |
| ***Ultrasonic procedures*** | | | | |
| Lim et al. 2016^42^ | Ferguson haemorrhoidectomy (*n*=25) vs ultrasonic scalpel (*n*=25) | Favours ultrasonic scalpel on POD3 (mean difference of 1.7; *P*<0.05) | Not reported | Acetaminophen, sitz baths |
| Shahmoradi et al. 2020^61^ | Ferguson haemorrhoidectomy (*n*=80) vs Harmonic scalpel (*n*=80) | Favours harmonic scalpel on POD1 (mean difference of 1.1; *P*=0.02) | Not reported | Not reported |
| Alhomoud et al. 2018^10^ | Conventional haemorrhoidectomy (*n*=25) vs Harmonic scalpel (*n*=25) | Favours harmonic scalpel on POD1 (mean difference of 1.4; *P*<0.05) | Not reported | Not reported |
| Shoukat et al. 2016^64^ | Bipolar diathermy (*n*=65) vs Harmonic scalpel (*n*=65) | Favours bipolar diathermy on POD1 (mean difference of 1.1; *P*<0.01) | Not reported | Not reported |
| ***Laser haemmorhoidoplasty*** | | | | |
| Longchamp et al. 2021^96^ | Laser haemorrhoidoplasty vs haemorrhoidal laser procedure (MA, 14 trials, 1570 patients) | Favours laser haemorrhoidoplasty (no timepoint; mean difference of 1.8; *P*<0.01) | Not reported | Not reported |
| Naderan et al. 2017^48^ | Laser intra-haemorrhoidal coagulation (*n*=30) vs Millian-Morgan haemorrhoidectomy (*n*=30) | Favours laser intra-haemorrhoidal coagulation on POD1 (mean difference of 1.2; *P*<0.01) | Not reported | Not reported |
| Poskus et al. 2020^53^ | Excisional haemorrhoidectomy (*n*=40) vs Laser haemorrhoidoplasty (*n*=40) vs sutured mucopexy (*n*=41) | Favours sutured mucopexy (no timepoint; *p*<0.01) | Not reported | Not reported |
| Shabahang et al. 2019^60^ | Laser haemorrhoidoplasty (*n*=40) vs Millian-Morgan haemorrhoidectomy (*n*=40) | NS at 0h and 6 weeks | Not reported | Not reported |
| ***Transanal haemorrhoidal dearterialization or haemorrhoidal artery ligation*** | | | | |
| Xu et al. 2019^105^ | Transanal dearterialization vs stapled haemorrhoidectomy (MA, 9 trials, 1077 patients) | NS | Not reported | Not reported |
| Emile et al. 2019^92^ | Transanal haemorrhoidal dearterialization vs stapled haemorrhoidopexy (MA, 6 trials, 554 patients) | Favours transanal dearterialization on POD1 (mean difference of 0.4; *p*<0.01) | Not reported | Not reported |
| Venturi et al. 2016^73^ | Transanal haemorrhoidal dearterialization (*n*=35) vs stapled haemorrhoidopexy (*n*=35) | NS on POD1 | Not reported | Acetaminophen, Codeine as needed |
| Lehur et al. 2016^40^ | Doppler-guided haemorrhoidal artery ligation (*n*=197) vs stapled haemorrhoidopexy (*n*=196) | Favours Doppler-guided haemorrhoidal artery ligation on POD1 (mean difference of 0.6; *P*<0.01) and during the second week (*P*=0.01) | Not reported | Not reported |
| Rorvik et al. 2020^56^ | Minimal open haemorrhoidectomy (*n*=45) vs transanal haemorrhoidal dearterialization (*n*=44) | Favours transanal haemorrhoidal dearterialization on POD1 to POD14 (mean difference of 3.5; *P*<0.01) | NS | Acetaminophen, ibuprofen, xylocaine gel, perianal infiltration, tramadol, morphine as needed |
| Carvajal et al. 2019^19^ | Excisional haemorrhoidectomy (*n*=20) vs haemorrhoid artery ligation with recto-anal repair (*n*=20) | Favours haemorrhoid artery ligation with recto-anal repair on POD0 to POD14 (mean difference of 2.0 on POD2; *P*<0.05) | Not reported | Acetaminophen, dexketoprofen, tramadol |
| Lee et al. 2021^39^ | LigaSure® assisted pile excision methods (*n*=40) vs Doppler-guided haemorrhoidal artery ligation with suture mucopexy (*n*=40) | Favours Doppler-guided haemorrhoidal artery ligation with suture mucopexy at 24h after surgery (mean difference of 2.9; *P*<0.01) | Not reported | Not reported |
| Tsunoda et al. 2017^70^ | Ultrasonic scalpel (*n*=22) vs transanal haemorrhoidal dearterialization with mucopexy (*n*=22) | Favours transanal haemorrhoidal dearterialization with mucopexy on POD1 (mean difference of 0.7; *P*<0.05) up to POD5 (*P*<0.05) | Not reported | Loxoprofen, flurbiprofen as needed |
| Aigner et al. 2016^6^ | Mucopexy (*n*=20) vs doppler-guided ligation of submucosal haemorrhoidal arteries and mucopexy (*n*=20) | NS | Not reported | Acetaminophen, lidocaïne cream 2%, piritramide as needed |
| Zhai et al. 2016^85^ | Suture fixation mucopexy (*n*=50) vs Doppler-Guided haemorrhoidal artery ligation (*n*=50) | NS | Not reported | Not reported |
| Trenti et al. 2019^69^ | Vessel-sealing device haemorrhoidectomy (*n*=41) vs transanal haemorrhoidal dearterialization with mucopexy (*n*=39) | NS on POD1 to POD15 | NS | Acetaminophen, NSAID, tramadol as needed |
| Leung et al. 2017^41^ | Tissue selective technique (*n*=40) vs transanal haemorrhoidal dearterialization (*n*=40) | NS on POD7 | NS | Acetaminophen, tramadol |
| Takada et al. 2021^67^ | Aluminum potassium sulfate and tannic acid injection with mucopexy (*n*=32) vs Doppler-guided transanal haemorrhoidal dearterialization procedure with mucopexy (*n*=22) | Favours aluminum potassium sulfate and tannic acid injection with mucopexy on POD5 and POD7 (*P*<0.05) | Not reported | Loxonine |
| ***Rubber band ligation*** | | | | |
| Dekker et al. 2021^91^ | Open or closed haemorrhoidectomy vs rubber band ligation (MA, 8 trials, 1208 patients) | Favours rubber band ligation (*P*<0.01) | Not reported | Not reported |
| Jin et al. 2021^35^ | Modified rubber band ligation (*n*=60) vs Millian-Morgan haemorrhoidectomy (*n*=60) | Favours modified rubber band ligation on POD1, POD3, POD7 and POD14 (*P*<0.01) | Not reported | Not reported |
| Brown et al. 2016^18^ | Haemorrhoidal arterial ligation (*n*=185) vs rubber band ligation (*n*=187) | Favours rubber band ligation on POD1 and 7 (mean difference of 1.2; *P*<0.01). NS on POD21 and 6 weeks postoperatively | Not reported | Not reported |
| Filgate et al. 2019^26^ | Haemorrhoid energy therapy (*n*=14) vs rubber band ligation (*n*=16) | Favours haemorrhoid energy therapy at 1h after surgery and on POD1 (mean difference of 1.7; *P*<0.05) | Not reported | Acetaminophen, ibuprofen as needed |
| Beg et al. 2017^16^ | Suction (*n*=30) vs forceps band ligation (*n*=30) | Favours suction at 1h (mean difference of 0.6, *P*=0.01) | Not reported | Not reported |
| **OTHER MODALITIES** | | | | |
| ***ERAS program*** | | | | |
| De Paula et al. 2022^23^ | Routine care (*n*=32) ERAS program (*n*=32) vs control (*n*=32) | NS from POD1 to POD30 | Favours ERAS program on POD30 for cumulative opioid consumption (*P*<0.01) | Not reported |
| ***Acupunture*** |  |  |  |  |
| Qin et al. 2020^99^ | Different acupuncture methods and related techniques (MA, 107 trials, 10,972 patients) | NS (no timepoint) | Not reported | Not reported |
| Wu et al. 2018^79^ | Electroacupuncture (*n*=36) vs control (*n*=36) | Favours electroacupuncture on POD 1 (mean difference of 0.4; *P*<0.05) | Not reported | Not reported |
| Yeh et Al. 2018^84^ | Electroacupuncture (*n*=39) vs control (*n*=41) | Favours electroacupuncture at 10h (mean difference of 1.8; *P*<0.01) | Not reported | NSAID, morphine routinely |
| Wang et al. 2020^75^ | Acupuncture (*n*=77) vs control (*n*=77) | Favours acupuncture on POD1 (mean difference of 1.8; *P*<0.01) and POD2 (*P*<0.01) | Favours acupuncture during the first 24h (*P*<0.01) | None |
