# Supplementary material for: PROSPECT guideline for haemorrhoid surgery: A systematic review and procedure-specific postoperative pain management recommendations
Source: Eur J Anaesthesiol Intensive Care. 2023 May 26;2(3):e0023. doi: 10.1097/EA9.0000000000000023 (PMC11783633; doi:10.1097/EA9.0000000000000023)
Supplement: Supplemental Digital Content [file ejaic-2-e0023-s003.docx]

**Table S2.** Summary of key results from studies evaluating pharmacological treatments, anaesthetic analgesic strategies, surgical procedures, and other modalities used to support the interventions that are not recommended for analgesic benefit in patients after haemorrhoid surgery. Bid, *bis in die*, twice a day; MA, meta-analysis; NS, not significant; NSAID, non-steroidal anti-inflammatory drugs; POD, postoperative day; SR, systematic review; tid, *ter in die,* three times a day**.**

| **Study** | **Study design** | | **Pain scores** | | **Cumulative opioid consumption** | | **Basic analgesia administered** | |
| --- | --- | --- | --- | --- | --- | --- | --- | --- |
| **PHARMACOLOGICAL INTERVENTIONS** | | | | | | | | |
| ***Oral metronidazole*** | | | | | | | | |
| Wanis et al. 2017^102^ | Oral metronidazole postoperatively for 3 days vs control (MA, 5 trials, 337 patients) | | NS on POD0 to POD7 | | Not reported | | Not reported | |
| Wilkie et al. 2021^77^ | Oral metronidazole 400mg tid (*n*=21) vs control (*n*=19) | | NS on POD1 to POD21 | | Not reported | | Acetaminophen, oral metronidazole, parecoxib, diclofenac, bilateral pudendal nerve block oxycodone as needed | |
| Lyons et al. 2017^97^ | Oral or topical metronidazole vs control (MA, 8 trials, 437 patients) | | Favours metronidazole on POD1 (mean difference of 1.4; *P*<0.01) and on POD7 (p  *P*<0.01) | | Not reported | | Not reported | |
| Xia et al. 2018^103^ | Oral or topical metronidazole vs control (MA, 9 trials, 523 patients) | | Favours oral and topical metronidazole on POD1 (mean difference of 1.3; *P*<0.01), POD7 (*P*<0.01) and POD14 (*P*<0.01) | | Not reported | | Not reported | |
| ***Topical atorvastatine*** | | | | | | | | |
| Ala et al. 2017^7^ | Atorvastatine 2% emulgel bid for 14 days (*n*=33) vs control (*n*=33) | | Favours atorvastatine on POD7 (mean difference of 1.5; *P*<0.01) and 14 (*P*=0.03). NS at 12h and on POD1, POD2 | | NS | | Acetaminophen as needed, magnesium hydroxyde as needed | |
| ***Topical baclofen*** | | | | | | | | |
| Ala et al. 2020^8^ | Topical baclofen (*n*=30) vs control (*n*=30) | | Favours baclofen on POD7 (mean difference of 1.3; *P*=0.01) and 14 (*P*=0.02) | | Not reported | | Acetaminophen | |
| ***Topical lidocaine combined with diclofenac*** | | | | | | | | |
| Linares-Gil et al. 2018^44^ | Topical lidocaine 2% plus diclofenac 0.5% (*n*=60) vs topical lidocaine 2% (*n*=60) | | Favours lidocaïne plus diclofenac from POD1 (mean difference of 2.8; *P*=0.03) to POD6 (mean difference of 3.7; *P*=0.01) | | Not reported | | Not reported | |
| ***Oral metronidazole and flavonoids*** | | | | | | | | |
| Rabelo et al. 2021^54^ | Metronidazole 400mg tid(*n*=17) vs flavonoids bid (coumarin 15 mg and troxerutin 90 mg; *n*=17) vs metronidazole 400mg tid and flavonoids bid (*n*=17) vs control (*n*=17) | | Favours metronidazole and flavonoids on POD7 (*P*<0.01), POD14 (*P*<0.01) and POD30 (*P*<0.01) when compared with metronidazole or control. Favours flavonoids over control and metronidazole on POD7 (mean difference of 3.0; *P*<0.01), POD14 (*P*<0.01) and POD30 (*P*<0.01) | | Not reported | | Not reported | |
| ***Sebacoyl dinalbuphine*** | | | | | | | | |
| Yeh et al. 2017^83^ | Sebacoyl dinalbuphine ester intramuscular injection 150 mg before surgery (*n*=109) vs control (*n*=112) | | Favours sebacoyl dinalbuphine ester on POD2 (mean difference of 0.4; *P*<0.01) and POD7 (*P*<0.01) | | Not reported | | Ketorolac | |
| **ANAESTHETIC AND ANALGESIC STRATEGIES** | | | | | | | | |
| ***Spinal anaesthesia*** | | | | | | | | |
| Borges et al. 2017^17^ | Perianal infiltration with ropivacaïne 0.75%, 20mL plus general anaesthesia (*n*=21) vs spinal anaesthesia (hyperbaric bupivacaine, 10mg) (n=19) | | Favours spinal anaesthesia at 30, 60, 90 and 120min (mean difference in VAS of 2.5; *P*<0.05) | | NS | | Acetaminophen, tramadol as needed | |
| Ruiz-Castro et al. 2017^57^ | Intrathecal hyperbaric bupivacaine 3mg with morphine 50µg (*n*=33) vs hyperbaric bupivacaine 5mg (*n*=33) | | Favours intrathecal hyperbaric bupivacaine 3mg with morphine 50µg on POD1 (mean difference of 1.8; *P*<0.01) | | Not reported | | Acetaminophen, dexketoprofen as needed | |
| ***Perianal infiltration*** |  | |  | |  | |  | |
| Hatami et al. 2022^27^ | Perianal infiltration of tramadol 2mg.kg^-1^ (*n*=30) vs bupivacaine 0.25%, 9mL (*n*=30) vs control (*n*=30) | | Favours tramadol vs control at 2h, 4h, 8h, 12h and 24h (mean difference of 0.7; *P*<0.05). NS for tramadol vs bupivacaine and bupivacaine vs control | | NS | | Ketorolac, pethidine | |
| Andrabi et al. 2021^13^ | Perianal infiltration with ropivacaine 0.75%, 20mL (*n*=25) vs control (*n*=25) | | Favours perianal infiltration (median difference of 1.7; *P*=0.05) | | Not reported | | Acetaminophen ibuprofen, ketorolac as needed | |
| Cho et al. 2020^22^ | Perianal infiltration with lidocaine 5% (*n*=53) vs control (*n*=58) | | NS at 1h, 6h and 24h | | Not reported | | Ketorolac | |
| Kanliöz et al. 2020^36^ | Perianal infiltration with liposomal bupivacaine 300mg (*n*=20) vs liposomal bupivacaine 300mg and local aloe vera 2% cream tid (*n*=20) vs perianal aloe vera 2% cream tid (*n*=20) vs control (*n*=32) | | Favours perianal infiltration liposomal bupivacaine and local aloe vera 2% cream over control at 24h (mean difference of 1.6; *P*<0.01) and 36h (*P*<0.01). Favours perianal aloe vera 2% cream over control at 24h (mean difference of 1.2; *P*<0.01) and 36h (*P*<0.01) | | Favours 300mg liposomal bupivacaine plus local aloe vera 2% cream vs control at 12h, 24h and 36h (*P*<0.01). Favours perianal aloe vera 2% cream vs control at 36h (*P*<0.01) | | Dexketroprofen as needed, pethidine as needed | |
| **SURGICAL PROCEDURES** | | | | | | | | |
| ***Millie-Morgan, Ferguson haemorrhoidectomy*** | | | | | | | | |
| Aibuedefe et al. 2021^88^ | Millian-Morgan, Ferguson haemorrhoidectomy vs different surgical techniques (MA, 26 trials, 3137 patients) | | Milligan-Morgan, Ferguson haemorrhoidectomy have the worst outcomes for postoperative pain. Laser, infrared photocoagulation and stapling reported lower pain-scores (no p-value reported) | | Not reported | | Not reported | |
| Balciscueta et al. 2021^89^ | Millian-Morgan haemorrhoidectomy vs different surgical techniques (MA, 29 trials, 3309 patients) | | Ultrasonic technique and circular haemorrhoidopexy associated with reduced pain | | Not reported | | Not reported | |
| Bhatti et al. 2016^90^ | Ferguson haemorrhoidectomy vs Milligan-Morgan haemorrhoidectomy (MA, 11 trials, 1326 patients) | | Favours Ferguson haemorrhoidectomy (*P*=0.01) | | Not reported | | Not reported | |
| Nikshoar et al. 2018^51^ | Infrared photocoagulation (*n*=20) vs Ferguson haemorrhoidectomy (*n*=20) | | Favours infrared photocoagulation on POD1 (mean difference of 3.4; *P*<0.05) | | Not reported | | Not reported | |
| Nikooiyan et al. 2016^50^ | Electrotherapy (*n*=60) vs Ferguson haemorrhoidectomy (n=60) | | Favours electrotherapy on POD1 (mean difference of 4.7; *P*<0.01) and POD7 (*P*<0.01) | | Not reported | | Not reported | |
| Vijayaraghavalu et al. 2021^74^ | Millian-Morgan haemorrhoidectomy plus lateral internal sphincterotomy (*n*=100) vs Millian-Morgan haemorrhoidectomy (*n*=100) | | Favours Millian-Morgan haemorrhoidectomy plus lateral internal sphincterotomy at 12h, 24h (mean difference of 2.2; *P*<0.01) and 48h (*P*<0.01) | | NS | | Tramadol | |
| Wang et al. 2018^101^ | Lateral internal sphincterotomy in excisional haemorrhoidectomy (MA, 10 trials, 1560 patients) | | Favours lateral internal sphinterotomy on POD1 (*P*<0.01) | | Not reported | | Not reported | |
| Fang et al. 2018^25^ | Milligan-Morgan haemorrhoidectomy plus purse string suture (*n*=34) vs procedure for prolapse and haemorrhoids (*n*=34) vs Milligan-Morgan haemorrhoidectomy (*n*=34) | | Favours Milligan-Morgan plus purse string suture and procedure for prolapse and haemorrhoids vs Milligan-Morgan on POD1 and POD7 (*P*<0.05). NS for Milligan-Morgan plus purse string suture vs procedure for prolapse and haemorrhoids | | Not reported | | Not reported | |
| Xianqing et al. 2021^81^ | Modified Park's submucosal haemorrhoidectomy (*n*=93) vs Millian-Morgan haemorrhoidectomy (*n*=93) | | Favours Millian-Morgan haemorrhoidectomy on POD1 (*P*<0.05). Favours modified Park's submucosal haemorrhoidectomy on POD7 (*P*<0.05). NS on POD3 and 14. | | Not reported | | Ketorolac | |
| Huang et al. 2021^32^ | Modified tissue selecting technique combined with complete anal canal epithelial retention surgery (*n*=87) vs Millian-Morgan haemorrhoidectomy (*n*=82) | | NS on POD3 | | Not reported | | Not reported | |
| Wu et al. 2019^78^ | Segmental resection (*n*=150) vs Milligan-Morgan haemorrhoidectomy (*n*=150) | | Favours segmental resection from POD1 to POD7 (mean difference of 2.9; *P*<0.05) | | Not reported | | Not reported | |
| Jia et al. 2021^34^ | High suspension and low incision surgery (*n*=123) vs Millian-Morgan haemorrhoidectomy (*n*=123) | | Favours high suspension and low incision surgery at 8h, 24h, 48h and 72h and at 1, 2 and 12 weeks (*P*<0.05) | | Not reported | | Not reported | |
| Zhai et al. 2021^86^ | Suture-fixation (n=60) vs Millian-Morgan haemorrhoidectomy (*n*=63) | | Favours suture-fixation on POD2 (mean difference of 3.5; *P*=0.03) | | Not reported | | Not reported | |
| **OTHER MODALITIES** | | | | | | | | |
| ***Postoperative medication checklist*** | | | | | | | | |
| Huang et al. 2020^33^ | Postoperative medication checklist (*n*=17) vs control (*n*=18) | | NS from POD1 to POD14 except for POD7 (mean difference of 1.3; *P*=0.03) | | Not reported | | Acetaminophen, ibuprofen, oral metronidazole, oxycodone | |
| ***Shuangjin haemorrhoid ointment and Beta-sodium aescinate*** | | | | | | | | |
| Zhao et al. 2021^87^ | Shuangjin haemorrhoid ointment and Beta-sodium aescinate (*n*=75) vs control (*n*=75) | | Favours Shuangjin and Beta-sodium on POD4 (mean difference of 0.6; *P*<0.05) and POD7 (mean difference of 0.9; *P*<0.05) | | Not reported | | Not reported | |
| ***Kangtai combined with Buzhong Yiqi*** | | | | | | | | |
| Lu et al. 2021^46^ | Chitosan gel (*n*=60) vs Kangtai ointment combined with modified Buzhong Yiqi decoction (*n*=60) | | Favours Kangtai combined with Buzhong Yiqi on POD14 (mean difference of 0.9; *P*<0.01) | | Not reported | | Tramadol | |
| ***Sitz bath*** | | | | | | | | |
| Shen et al. 2017^63^ | Sitz bath with warm water (*n*=155) vs sitz bath with Xiaozhi decoction (*n*=155) | | Favours Xiaozhi on POD2 (mean difference of 0.3; *P*=0.02), POD7 and POD14 (*P*<0.01) | | Favours Xiaozhi on POD2 (*P*=0.01), 7 and 14 (*P*<0.01) | | Acetaminophen, metronidazole tramadol as needed | |
| ***Karamardadi yoga*** | | | | | | | | |
| Hegana et al. 2016^29^ | Karamardadi yoga 1500mg tid (*n*=30) vs sodium diclofenac 50mg tid (*n*=30) | | NS on POD1 to POD3 | | Not reported | | Not reported | |
| ***Venoplant*** | | | | | | | | |
| Schiano et al. 2017^59^ | Venoplant (Diosmin 300 mg, coumarin 32 mg, and triterpenes in C. asiaticaat 15 mg) tid (*n*=91) vs control (*n*=91) | Favours venoplant on POD15 (mean difference of 1.0) and POD30 (*P*<0.05). NS on POD7 | | Not reported | | Acetaminophen | |  |
| ***Flavonoids*** | | | | | | | | |
| Chiaretti et al. 2020^21^ | Flavonoids tablets 300mg bid and 3g of intra-anal flavonil (*n*=73) vs calcium tablets 60mg bid (*n*=9) vs control (*n*=12) | | Favours flavonoid vs control and calcium vs control from POD1 (*P*<0.01) to 6 weeks (*P*=0.04) | | Not reported | | Not reported | |
