# Supplementary material for: PROSPECT guideline for haemorrhoid surgery: A systematic review and procedure-specific postoperative pain management recommendations
Source: Eur J Anaesthesiol Intensive Care. 2023 May 26;2(3):e0023. doi: 10.1097/EA9.0000000000000023 (PMC11783633; doi:10.1097/EA9.0000000000000023)
Supplement: Supplemental Digital Content [file ejaic-2-e0023-s004.docx]

**Table S3.** List of articles excluded and reasons for exclusion.

| ***Study*** | **Comparators** | ***Reason for exclusion*** |
| --- | --- | --- |
| Abiodun et al. 2020 | Endoscopic injection sclerotherapy vs endoscopic rubber band ligation | No pain outcomes reported |
| Ahmed et al. 2021 | Harmonic scalpel vs Milligan Morgan technique | No pain outcomes reported |
| Aiyanna et al. 2020 | More extensive mucosal resection | No pain outcomes reported |
| Aljabery et al. 2020 | Lord dilatation vs lateral internal sphincterotomy | No pain outcomes reported |
| Alshreef et al. 2017 | Haemorrhoidal Artery Ligation vs Rubber Band Ligation | No pain outcomes reported |
| Altomare et al. 2017 | Standard (PPH Ethicon Endosurgery) stapled haemorrhoidopexy vs larger volume circular stapler (EEA Covidien) | No pain outcomes reported |
| Alvandipoor et al. 2019 | Effect of Diosmin on post-hemorrhoidectomy pain. | Article not in English |
| Asif et al. 2020 | Examine the efficacy of 0.2% topical glyceryl trinitrate vs control group | No pain outcomes reported |
| Bakhtawar et al. 2017 | Mlligan-Morgan haemorrhoidectomy vs rubber band ligation technique | Abstract only |
| Butt et al. 2018 | Open haemorrhoidectomy with vs without lateral internal sphincterotomy | No pain outcomes reported |
| Chen et al. 2018 | Silicate-based wound dressing (dermfactor) vs control group | No pain outcomes reported |
| Corsale et al. 2018 | Flavonoids mixture (diosmin, troxerutin, rutin, hesperidin, quercetin) to reduce bleeding from I-III degrees hemorrhoidal disease | Not procedure specific |
| Ding et al. 2019 | Virtual reality distraction (n=91) vs control (n=91) | Not procedure specific |
| Du et al. 2019 | Comparison of surgical procedures | No pain outcomes reported |
| Elahabadi et al. 2021 | Rectal acetaminophen vs diclofenac | Article not in English |
| Fadel et al. 2021 | Role of Pudendal Nerve Block in Colorectal Surgery (SR) | Not procedure specific |
| Farag et al. 2016 | Caudal administration of bupivacaine 0.25% in 20 ml vs tramadol 1 mg/kg in 20 ml vs tramadol 2 mg/kg in 20 ml | No pain outcomes reported |
| Fu et al. 2016 | Ultrasonic knife combined with peel bridge lateral transfer vs traditional external dissection and internal ligation | Article not in English |
| Geary et al. 2020 | Task trainers in haemorrhoidectomy | Not procedure specific |
| Genova et al. 2019 | Transanal hemorrhoidal dearterialization vs Milligan-Morgan hemorrhoidectomy | No pain outcomes reported |
| Giarratano et al. 2018 | Transanal hemorrhoidal dearterialization vs stapled hemorrhoidopexy | No pain outcomes reported |
| He et al. 2017 | Ruiyun procedure for hemorrhoids (RPH) vs RPH with the simplified Milligan-Morgan hemorrhoidectomy | No pain outcomes reported |
| He et al. 2017 | PPH with the simplified Milligan-Morgan surgery vs Ruiyun procedure for hemorrhoids with the simplified Milligan-Morgan surgery | Article not in English |
| Hosseini et al. 2018 | Effects of Stapled Hemorrhoidopexy on Anorectal Function | Not procedure specific |
| Huang et al. 2016 | Tying, binding and fixing operation' vs Doppler ultrasound-guided hemorrhoidal artery ligation | No pain outcomes reported |
| Huang et al. 2020 | Shaobei injection vs elastic band ligation | Article not in English |
| Hussein et al. 2020 | Laser surgery vs the traditional type of surgery | Not procedure specific |
| Jiang et al. 2017 | PPH plus single-purse suture group vs PPH plus multipoint umbrella suture | No pain outcomes reported |
| Jinjil et al. 2018 | Spinal anaesthesia vs local perianal block | No pain outcomes reported |
| Khan et al. 2019 | Daflon vs band ligation | Abstract only |
| Khowaja et al. 2019 | Open hemorrhoidectomy with or without lateral internal sphincterotomy | Abstract only |
| Kibret et al. 2021 | Prevalence and associated factors of hemorrhoids | Not procedure specific |
| Kilonzo et al. 2018 | Stapled Haemorrhoidopexy vs traditional Excisional Surgery | No pain outcomes reported |
| Kovalev et al. 2021 | Standard of care and the rehabilitation program, including intravenous ozone therapy, rectal laser therapy, recto-tibial myostimulation, and biofeedback therapy vs control group | Article not in English |
| Kumar et al. 2021 | Lithotomy vs Prone Position for Perianal Surgery | Not procedure specific |
| Lashari et al. 2020 | Received 0.2% of topical glyceryl trinitrate ointment vs control group | No pain outcomes reported |
| Lehmann et al. 2020 | Long-term functional results after excisional haemorrhoidectomy | Not procedure specific |
| Li et al. 2016 | Chinese medicine fumigation treatment vs control group | Abstract only |
| Lin et al. 2016 | Moxa box moxibuste point Baihuanshu point, Yao point and Huiyang point vs intramuscular injection lidocaine diclofenac sodium 75 mg. | Abstract only |
| Lin et al. 2017 | Electroacupunture at point Neiguan (PC 6), Shenmen (HT 7), Shangliao (BL 31) and Ciliao (BL 32) vs electroacupunture at point Shangliao (BL 31) and Ciliao (BL 32) vs control group | Article not in English |
| Liu et al. 2017 | Meta-analysis of Doppler ultrasonic guided hemorrhoid artery ligation | No pain outcomes reported |
| Long et al. 2018 | Electroacupunture group vs auricular point sticking group vs combined group | Article not in English |
| Long et al. 2019 | Electroacupuncture group vs control group | Article not in English |
| Long et al. 2019 | Electroacupuncture with dilatational wave, 2 Hz/100 Hz in frequency vs electroacupuncture with continuous wave, 2 Hz in frequency vs electroacupuncture with continuous wave, 100 Hz in frequency | Article not in English |
| Lu et al. 2019 | Aescuven forte tablets vs control group | Article not in English |
| Mahmood et al. 2016 | Open vs closed haemorrhoidectomy | No pain outcomes reported |
| Massoumi et al. 2020 | Postoperative physician phone calls | Not procedure specific |
| Medkova et al. 2019 | Surgical (thrombectomy or local excision of external haemorrhoids) vs conservative treatment | Article not in English |
| Mehta et al. 2021 | 1 ml of 0.5% hyperbaric bupivacaine with 20µg fentanyl(0.4ml) vs 1ml of 0.5% hyperbaric bupivacaine with 200µg butorphanol(0.2ml) and normal saline(0.2ml) for perianal surgeries | Not procedure specific |
| Memarbashi et al. 2021 | Arnebia euchroma ointment vs control group | Article not in English |
| Mengal et al. 2017 | Stapled vs conventional hemorrhoidectomy | No pain outcomes reported |
| Mohamedahmed et al. 2020 | Local anaesthesia (bilateral pudendal nerve block, perianal block, local infiltration and sedation) vs spinal anaesthesia | Not procedure specific |
| Mohammed et al. 2017 | Conventional vs stapled hemorrhoidectomy | No pain outcomes reported |
| Mongelli et al. 2021 | Spinal anaesthesia with or without the pudendal nerve block | No pain outcomes reported |
| Perivolitis et al. 2019 | Ligation and hemorrhoidopexy vs ultrasound guided ligation of hemorrhoidal arteries | No pain outcomes reported |
| Popovtsev et al. 2021 | Hemorrhoidal artery ligation with or without Doppler navigation | Article not in English |
| Rasool et al. 2020 | Diathermy haemorrhoidectomy vs Milligan-Morgan haemorrhoidectomy | No pain outcomes reported |
| Rodoman et al. 2017 | Haemorrhoidal Artery Ligation Recto Anal Repair vs closed hemorrhoidectomy with linear stapler | Article not in English |
| Rodriguez-Wong et al. 2016 | Topical diltiazem vs control group | Article not in English |
| Rodriguez-Wong et al. 2019 | Topical diltiazem vs control group | Article not in English |
| Rotigliano et al. 2020 | Perianal block with ropivacaine as a supplement to anaesthesia in proctological surgery vs control group | Not procedure specific |
| Rozsos et al. 2021 | Use of laser fibers vs use of microwave rigid antenna | Article not in English |
| Saeed et al. 2017 | Milligan-Morgan haemorrhoidectomy vs rubber band ligation | Abstract only |
| Salgueiro et al. 2021 | Polidocanol Foam Sclerotherapy vs Rubber Band Ligation | No pain outcomes reported |
| Salimi et al. 2017 | Carbamazepine used preemptively vs control group | No pain outcomes reported |
| Schreckenbach et al. 2016 | Proctologic surgery participation by residents | Not procedure specific |
| Shafi et al. 2021 | 5% phenol almond oil vs polidocanol sclerotherapy | No pain outcomes reported |
| Sharma et al. 2021 | Local anaesthetic vs control | No pain outcomes reported |
| Shehata et al. 2019 | Doppler-Guided Hemorrhoidal Artery Ligation vs Rubber Band Ligation | No pain outcomes reported |
| Soltany et al. 2019 | Hemorrhoidectomy with or without sphincterotomy | Abstract only |
| Song et al. 2017 | Electroacupuncture vs control group | Article not in English |
| Song et al. 2019 | Preoperative electroacupuncture vs control group | Article not in English |
| Sotnikov et al. 2019 | Hepatrombin H® ointment and excision of external hаemorrhoids vs control group | Article not in English |
| Stessel et al. 2019 | Metamizole vs ibuprofen at home after day case surgery | Not procedure specific |
| Stessel et al. 2021 | Predictors of quality of recovery after painful day case surgery | Not procedure specific |
| Titov et al. 2016 | Doppler-assisted dearterialization of internal hemorrhoids with mucopexy vs hemorrhoidectomy using harmonic scalpel | Article not in English |
| Trenti et al. 2017 | Distal Doppler-guided transanal hemorrhoidal dearterialization with mucopexy vs conventional hemorrhoidectomy | No pain outcomes reported |
| Van Backer et al. 2018 | Preoperative oral acetaminophen and gabapentin vs control group | Not procedure specific |
| Van Tol et al. 2018 | Comparison of outcomes for hemorrhoidal disease | No pain outcomes reported |
| Venara et al. 2018 | Transanal Doppler-guided hemorrhoidal artery ligation with mucopexy vs circular stapled hemorrhoidopexy | No pain outcomes reported |
| Wang et al. 2021 | Perioperative intravenous S-ketamine for acute postoperative pain | Not procedure specific |
| Wen et al. 2017 | Electroacupuncture group vs catgut implantation group | Article not in English |
| Wu et al. 2016 | Electroacupuncture vs control group | Article not in English |
| Xia et al. 2020 | Local anaesthesia vs regional or general anaesthesia in excisional haemorrhoidectomy | No pain outcomes reported |
| Xu et al. 2016 | Transanal hemorrhoidal dearterialization with mucopexy vs open hemorrhoidectomy | No pain outcomes reported |
| Xu et al. 2016 | Nerve‐related acupoints electrical stimulation 1h after Milligan‐Morgan hemorrhoidectomy vs control group | No pain outcomes reported |
| Xu et al. 2018 | Caudal anaesthesia using 18 ml 0.3% ropivacaine plus 2 ml normal saline vs 18 ml 0.3% ropivacaine plus 2 ml 1 µg/kg dexmedetomidine. | No pain outcomes reported |
| Yamamoto et al. 2020 | Postoperative outcomes of hemorrhoidectomy in the elderly (>=75 years old) vs non-elderly patients (<75y old) | No pain outcomes reported |
| Yang et al. 2016 | Potassium permanganate umigation vs control group | Abstract only |
| Yang et al. 2020 | Laparoscopic integral pelvic floor/ligament repair alone vs laparoscopic integral pelvic floor/ligament repair combined with procedure for prolapse and hemorrhoids | No pain outcomes reported |
| Yao et al. 2018 | Transanal hemorrhoidal dearterialization vs stapled hemorrhoidectomy | No pain outcomes reported |
| Yasser et al. 2020 | Caudal epidural vs pudendal nerve block | Not procedure specific |
| Zarbaliyev et al. 2021 | The relationship between smartphone use in the lavatory and hemorrhoidal disease | Not procedure specific |
| Zeng et al. 2021 | Kangfuxin solution fumigation bath vs warm water sitz bath | No pain outcomes reported |
| Zhang et al. 2020 | Procedure for prolapse and hemorrhoids vs Milligan-Morgan hemorrhoidectomy vs tissue-selecting therapy stapler | No pain outcomes reported |
| Zhao et al. 2016 | Jiawei shaoyao gancao decoction vs piles‐healthy slice | Abstract only |
| Zhu et al. 2020 | Titanoreine vs control group | No pain outcomes reported |
